# GenMap: ultra-fast computation of genome mappability

**DOI:** 10.1093/bioinformatics/btaa222

**Published:** 2020-04-04

**Authors:** Christopher Pockrandt, Mai Alzamel, Costas S Iliopoulos, Knut Reinert

**Affiliations:** b1 Center for Computational Biology, School of Medicine; b2 Department of Biomedical Engineering, Johns Hopkins University, Baltimore, MD, USA; b3 Department of Computer Science and Mathematics, Freie Universität Berlin; b4 Department of Computational Molecular Biology, Max Planck Institute for Molecular Genetics, Berlin, Germany; b5 Department of Informatics, King’s College London, London, UK; b6 Department of Computer Science, King Saud University, Riyadh, Saudi Arabia

## Abstract

**Motivation:**

Computing the uniqueness of *k*-mers for each position of a genome while allowing for up to *e* mismatches is computationally challenging. However, it is crucial for many biological applications such as the design of guide RNA for CRISPR experiments. More formally, the uniqueness or (*k*, *e*)-mappability can be described for every position as the reciprocal value of how often this *k*-mer occurs approximately in the genome, i.e. with up to *e* mismatches.

**Results:**

We present a fast method GenMap to compute the (*k*, *e*)-mappability. We extend the mappability algorithm, such that it can also be computed across multiple genomes where a *k*-mer occurrence is only counted once per genome. This allows for the computation of marker sequences or finding candidates for probe design by identifying approximate *k*-mers that are unique to a genome or that are present in all genomes. GenMap supports different formats such as binary output, wig and bed files as well as csv files to export the location of all approximate *k*-mers for each genomic position.

**Availability and implementation:**

GenMap can be installed via bioconda. Binaries and C++ source code are available on https://github.com/cpockrandt/genmap.

## 1 Introduction

Analyzing data derived from massively parallel sequencing experiments often depends on the process of genome assembly via resequencing; namely, assembly with the help of a reference sequence. In this process, a large number of reads derived from a DNA donor during these experiments must be mapped back to a reference sequence, comprising a few gigabases to establish the section of the genome from which each read originates. An extensive number of short-read alignment techniques and tools have been introduced to address this challenge emphasizing different aspects of the process (Fonseca *et al.*, 2012). Given a set of reads of some fixed length *k* the process of resequencing depends heavily on how mappable a genome is. Thus, for every substring of length *k* in the sequence, we want to count how many times this substring appears in the sequence while allowing for a small number *e* of errors. In other terms, mappability is a measure of how unique or repetitive regions in the genome are and is closely related to mapping. The concept of mappability for sequence analysis was introduced by Koehler *et al.* (2011), taken up again and later formalized by Derrien *et al.* (2012) (see also Antoniou *et al.*, 2009).

While *k*-mer counting became extremely popular in the last years, searching for *k*-mers with low occurrences does not meet the needs of many applications. Sequencing errors and variations, such as SNPs, require not only the *k*-mer to be unique or rare enough but also close matches of this *k*-mer, i.e. all *k*-mers with a certain number of mismatches have to be considered as well. Since the number of *k*-mers to be considered grows exponentially in the number of mismatches, *k*-mer counters are infeasible for this problem.

The suite GEM-Tools (Marco-Sola *et al.*, 2012) includes a program to compute the mappability for arbitrary *k*-mers and number of mismatches. It is the most common and advanced algorithm to compute the mappability of entire genomes. To improve its performance, it offers a heuristic mode leading only to an approximation of the mappability. Nevertheless, it is not feasible to compute many instances of biological relevant *k*-mer sizes and number of errors.

In the following paragraph, we give a formal definition of the problem, present our algorithm in the next section and compare it to GEM. Our algorithm does not rely on heuristics and outperforms GEM even in its heuristic mode by far.

**Definition [(*k*, *e*)-mappability and (*k*, *e*)-frequency].** Given a string *T* of length *n*, the (*k*, *e*)-frequency counts occurrences of every single *k*-mer in *T* with up to *e* errors. We denote the *k*-mer starting at position *i* in *T* as *T_i_*. The values are stored in a frequency vector *F* of length n−k+1 such that

$$F[i]=|\{j|D(T_{i},T_{j})\leq e,1\leq j\leq n-k+1\}|,$$

where $D(T_{i},T_{j})$ denotes the distance of two *k*-mers given a metric such as Hamming or Edit distance. Its elementwise multiplicative inverse is called the (*k*, *e*)-mappability and stored in a mappability vector *M* with M[i]=1/F[i] for 1≤i≤n−k+1.

**Fig. 1.**
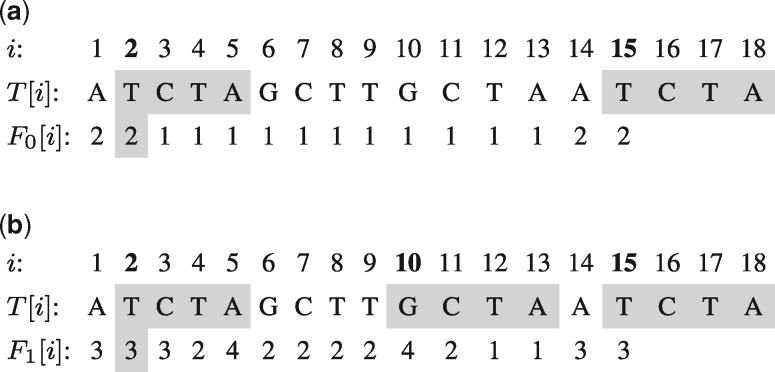
(*k*, *e*)-frequency vectors *F_e_* for *k*=4 and e∈{0,1} on the same sequence. A frequency of 1 indicates that the *k*-mer starting at that position in the text is unique in the entire sequence without errors, respectively, with up to one mismatch. (**a**) (4, 0)-frequency and (**b**) (4, 1)-frequency

A mappability value of 1 represents a unique *k*-mer, a mappability value close to 0 indicates a *k*-mer occurring in repetitive regions. Figure 1 gives an example for the (4, 0)- and (4, 1)-frequency of a given nucleotide sequence considering Hamming distance.

Since, for some applications, an exact computation of mappability is favorable, we propose a new algorithm that is not only faster than previous ones but also exact, i.e. does not rely on heuristics. Mappability can not only be used straightforward to retrieve information on the repetitiveness of the underlying data. In this paper, we will also illustrate that it can be used to find marker sequences that allow distinguishing similar strains of the same species, as well as separate strains by groups sharing common *k*-mers.

## 2 Materials and methods

Before we present our algorithm, we give an overview of the approach of Derrien *et al.* to compute the (*k*, *e*)-mappability. For reasons of clarity, we consider computing its inverse, the (*k*, *e*)-frequency and neglect searching the reverse strand throughout this paper. To consider the reverse strand, each *k*-mer has simply to be searched by its reverse complement leading to a doubling of the running time. Furthermore, we consider Hamming distance, if not stated otherwise. It can be applied to other distance metrics such as Edit distance as well.

To achieve a feasible running time Derrien *et al.* implemented a heuristic. First, they initialize the frequency vector with 0 s and perform a linear scan over the text (see Algorithm 1). Then each *k*-mer *T_i_* is searched with *e* errors in an FM index and the number of occurrences is stored in *F*[*i*]. If the count value exceeds some user-defined threshold parameter *t*, the locations of these occurrences are located. Let *j* be such a location. Since *T_i_* has a high frequency, i.e. F[i]>t and $D(T_{i},T_{j})\leq e$, it is likely that *T_i_* and *T_j_* share common approximate matches. Hence, *F*[*j*] is assigned the frequency value *F*[*i*]. To speed up the computation, *k*-mers that already have frequency values assigned by this heuristic step are skipped during the scan over the text. If a position *j* is located multiple times as an approximate match of a repetitive *k*-mer, *F*[*j*] is assigned the maximum frequency of all these *k*-mers to avoid underestimating the frequency value *F*[*j*].

Algorithm 1. Inexact algorithm to compute the (*k*, *e*)-frequency by Derrien *et al.*

1:  **procedure** inexact_frequency (T,k,e,t)

2:   F[1..|T|−k+1]←{0}

3:   **for**i=1,…,|F|**do**

4:    **if**F[i]=0**then**

5:     P←**approximate matches with** *e* **errors**

6:     F[i]←|P|

7:     **if**|P|>t**then**

8:      **for**j∈P**do**

9:      F[j]←max(F[j],|P|)

10:   **return** *F*

Their experiments on chromosome 19 of the human genome with *t*=7 show that almost 90% of the 50-mers with a frequency of 3 are correct, for 50-mers with frequency values between 8 and 12 only 75% are correct (similar errors for *Caenorhabditis elegans* with *t*=6). This can be led back to an overestimation of rather unique *k*-mers.

We now present a fast and exact algorithm to compute the (*k*, *e*)-frequency. Similar to the algorithm implemented in GEM we scan over the text *T* while searching and counting the occurrences of each *k*-mer *T_i_* for 1≤i≤n−k+1 with up to *e* errors in an index on *T*. In contrast to GEM, we improve the running time by reducing redundant searches with three major improvements which we introduce in the following.

### 2.1 Approximate string matching using optimum search schemes

When searching a *k*-mer in a string index, it is searched character by character. Unidirectional indices only support extending characters into one direction, either to the left or to the right, while bidirectional indices support searching into both directions in any arbitrary order (Lam *et al.*, 2009). To search for every possible approximate match within the given error bound, backtracking is performed. This leads to exponential running time in the number of errors. Especially allowing for errors at the beginning of the *k*-mer, i.e. branching at the topmost nodes in the backtracking tree is expensive. Hence, we use optimum search schemes (Kianfar *et al.*, 2018) when searching each *k*-mer, a sophisticated search strategy that reduces the number of search steps performed in the index while still searching for all possible approximate matches.

Optimum search schemes are based on a framework by Kucherov *et al.* called search schemes that allows formalizing search strategies in a bidirectional index (Kucherov *et al.*, 2016). The sequence to be searched is split into *p* pieces and searched by certain combinations of the pieces in the index while trying to reduce the number of search steps performed in the index.

Formally, a search is a triplet S=(π,L,U) of integer strings each of length *p*. *π* is a permutation of the numbers {1,2,…,p} indicating the order in which the pieces are searched. Starting from an arbitrary piece π[0] the subsequent pieces need to be adjacent to the previously searched pieces. *L* and *U* are non-decreasing integer strings indicating the lower and upper bound of errors. After the piece π[i] is searched a total number of errors from L[i] to U[i] must have been spent. A set of searches that covers all possible error distributions with *e* errors and *p* pieces forms a search scheme. As a result, the number of errors allowed in the first pieces of each search is reduced which speeds up approximate string matching. By performing multiple searches starting with different pieces, it is guaranteed that all possible error distributions among the pieces are covered.

**Fig. 2.**
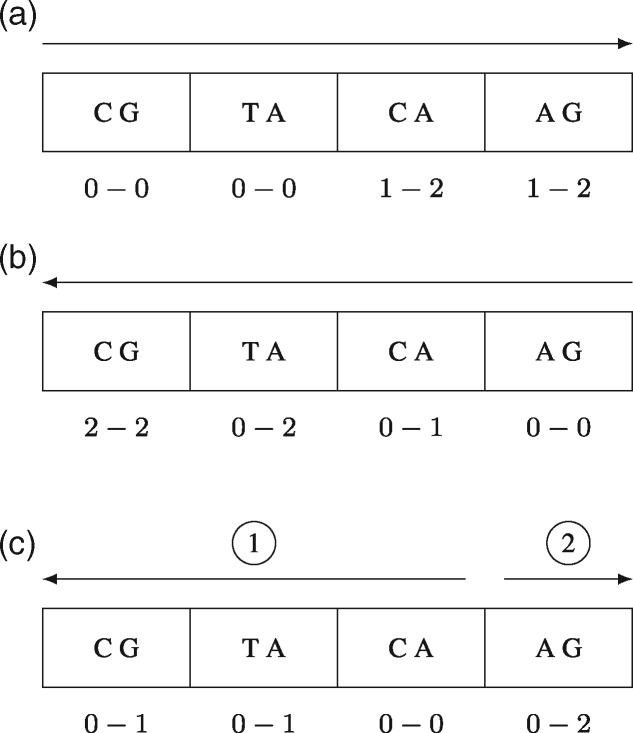
The optimum search scheme for two mismatches consists of three searches with four pieces each. The arrows indicate in which order the pieces are searched. The error bounds below each part are cumulative bounds, i.e. the minimum number of errors that *must*, respectively, the maximum number of errors that *can* be spent until searching the end of the corresponding piece. Illustrated for searching the 8-mer CGTACAAG. The forward search covers the error distributions 0010, 0011, 0020, the backward search covers 2000, 1100, 0200, 1010, 0110 and the bidirectional search 0000, 0001, 0002, 1000, 1001, 0100, 0101. (**a**) Forward search: *S_fwd_*=(1234, 0011, 0022); (**b**) backward search: *S_bwd_*=(4321, 0002, 0122) and (**c**) bidirectional search: *S_bi_*=(3214, 0000, 0112)

Optimum search schemes are search schemes that are optimal under certain constraints, i.e. the number of backtracking steps in an index over all searches are minimized while still covering all error distributions. Figure 2 illustrates the optimum search scheme for *e*=2 errors, p=e+2 pieces and up to three searches.

A bidirectional index is required to enable to start a search with a middle piece as illustrated in Figure 2c. To improve the overall running time of the index-based search, we use a fast implementation of bidirectional FM indices based on enhanced prefixsum rank (EPR) dictionaries (Pockrandt *et al.*, 2017).

### 2.2 Adjacent *k*-mers

**Fig. 3.**
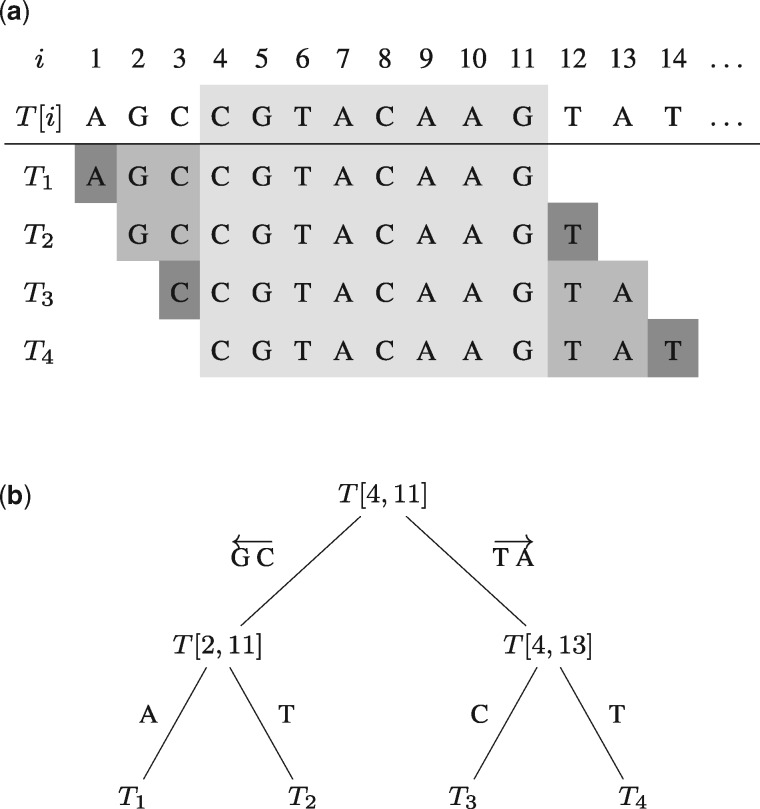
Searching *s* overlapping *k*-mers using optimum search schemes for the infix and extending it using backtracking. Illustrated for *k*=11 and *s*=4. (**a**) First, the common overlap (light gray) is searched using optimum search schemes. Second, the search of *T*_1_ and *T*_2_ is continued recursively by extending the previously identified approximate matches of the infix in the index by GC to the left (allowing for the remaining number of errors; medium gray). *T*_1_ and *T*_2_ are then retrieved separately by backtracking in the index by one character to the left and one character to the right (allowing for an error, if any left; dark gray). *T*_3_ and *T*_4_ are extended analogously in a recursive manner. (**b**) The same strategy presented as a backtracking tree. It is traversed for all occurrences reported by the search of the infix *T*[4, 11] using optimum search schemes. Each edge also has to account for remaining errors, i.e. approximate string matching is performed using backtracking

Adjacent *k*-mers in *T* are highly similar, since they have a large overlap. Hence, we do not search for every *k*-mer separately. Consider the adjacent *k*-mers $T_{j},T_{j+1},\ldots ,T_{j+s-1}$ for some integer s≤k−e+1 which all share the common sequence T[j+s−1..j+k−1] of length at least *e*. Since we already need to allow for up to *e* errors in their common sequence when searching each *k*-mer, this infix should only be searched once. Thus, we start searching this infix using optimum search schemes and extend it afterwards to retrieve the occurrences for each *k*-mer separately using backtracking, allowing for the remaining number of errors not spent in the search of the infix. Since the extension is performed in both directions, a bidirectional index is required. Figure 3a illustrates this approach.

To further reduce the number of redundant computations, the set of overlapping *k*-mers is recursively divided into two sets of *k*-mers of roughly equal size that each share a larger common overlap among each other. This overlap is then searched using backtracking before the next recursive partitioning of *k*-mers. The recursion ends when a single *k*-mer is left and the number of occurrences can be reported and summed up, or no hits are found. The recursive extension is shown in Figure 3b. Note that there are two recursions involved: subdividing the set of *k*-mers and backtracking in each recursion step. Hence, the same partitioning steps and backtracking steps have to be performed for each set of preliminary matches represented by suffix array ranges in the FM index.

The question remains on how to combine the improvements of Sections 2.1 and 2.2, i.e. how to choose *s*, the number of adjacent *k*-mers that are searched together starting with their common sequence using optimum search schemes. On the one hand, approximate string matching using optimum search schemes is more efficient than simple backtracking; hence, a longer common infix is favorable. On the other hand, a longer common infix means fewer adjacent *k*-mers are searched at once which leads to more redundant search steps due to the high similarity of overlapping *k*-mers. GenMap chooses *s* according to the following equation derived from optimal values that were determined experimentally on different genomes such as the human and barley genome [see Pockrandt (2019) for details]. clamp(v,l,r) returns *v* if it lies within the range, i.e. l≤v≤r, and returns *l* or *r* if it is less or greater

$$s=\{\begin{array}{cc} \left\lfloor k\cdot 0.7\right\rfloor & ,\;e=0\\ \left\lfloor k\cdot \left(\text{clamp}\left(\frac{k}{100},0.3,1.0\right)\cdot 0.7^{e}\right)\right\rfloor & ,\;\text{otherwise} \end{array}.$$

### 2.3 Skipping redundant *k*-mers

Finally, we avoid searching the same *k*-mer multiple times. Especially *k*-mers from repeat regions may occur many times without errors in the text. Since they all share the same frequency value, it should be avoided to compute it more than once. Hence, after searching and counting the occurrences of a *k*-mer, we locate the positions of the exact matches and set all their frequency values in *F* accordingly.

We observed that this strategy leads to longer runs of frequency values forwarded to positions with uncomputed frequency values. When forwarded frequency values of previously counted *k*-mers are encountered during the scan over the text, they are skipped.

## 3 Results

### 3.1 Benchmarks

At first, we compare the running times for computing the frequency on the human genome for different lengths and errors based on Hamming distance. We ran GEM in its exact mode as well as in its heuristic mode. For the latter, the authors recommend *t*=7. Table 1 compares the running times for shorter *k* that are of interest for applications such as identifying marker sequences, presented in Section 3.2. Table 2 shows typical instances used for applications in read mapping based on a typical Illumina read length. Even though longer Illumina read lengths are more common these days, we choose a shorter read length, since the frequency is easier to compute for longer *k*-mers.

**Table 1.** Running times for computing the frequency of the human genome (GRCh38) using 16 threads

Tool	(36, 0)	(24, 1)	(36, 2)	(50, 2)	(75, 3)
Instances are taken from the experiments by Derrien *et al.* (2012)					
GEM exact	5 h 10 m	N/A	N/A	N/A	N/A
GEM heuristic	23 m	N/A	7 h 11 m	5 h 50 m	4 h 26 m
GenMap	3 m	23 m	1 h 19 m	42 m	1 h 27 m
Tool	(101, 0)	(101, 1)	(101, 2)	(101, 3)	(101, 4)
Typical Illumina read length with growing number of mismatches					
GEM exact	44 m	7 h 28 m	7 h 34 m	7 h 45 m	8 h 8 m
GEM heuristic	28 m	2 h 40 m	3 h 17 m	3 h 31 m	3 h 49 m
GenMap	2 m	7 m	17 m	46 m	2 h 42 m

*Note*: Timeouts of 1 day are represented as N/A.

**Table 2.** (30, 2)-mappability on four strains of *E.coli* assigned to the phylogenetic group B1 based on the known marker genes by Clermont *et al.*

	All *k*-mers			Non-adjacent *k*-mers		
Strain	Unique	Pseudo	∅ Dist.	Unique	Pseudo	∅ Dist.
IAI1	171 942	4992	27 ± 627	1829	81	2476 ± 5560
SE11	305 439	10 365	15 ± 447	2356	176	1942 ± 4708
11128	260 305	40 101	20 ± 953	2494	685	2049 ± 9517
11368	434 033	108 968	13 ± 912	3142	1116	1674 ± 10 592

*Note*: We computed the mean distance of the unique marker sequences and their standard deviation.

For all computed instances, GenMap is faster than GEM. Compared to the approximate mode, we are almost a magnitude faster for a smaller number of errors, but for 4 errors, the heuristic of GEM pays off and is almost as fast as our algorithm. Interestingly, the increase of the running time of GEM in its exact mode gets smaller with more errors. For 101-mers with 1–4 errors, the running time is always about 7–8 h, nonetheless GenMap is still faster by a factor from 3 of up to 64 (4 and 1 errors). Even when searching without errors where no backtracking has to be performed, our tool is faster by a factor of 20–100 (for 101-mers and 36-mers). The most noticeable improvement is achieved for short *k*-mers. Derrien *et al.* point out that their algorithm is not suitable for small *k* and completely unfeasible for *k*<30 without its heuristic which is reflected by our benchmarks, whereas GenMap can handle these instances easily. GEM takes significantly longer, often does not even terminate within 24 h on 16 threads.

GenMap is also faster than GEM when computing the frequency of small genomes like *Drosophila melanogaster*. Since smaller genomes are generally less challenging, we omit the benchmarks here. For the human genome, the memory consumption of GenMap is about 9 GB (using a bidirectional FM index with EPR dictionaries and a suffix array sampling rate of 10), while GEM takes up 4.5 GB (using an unspecified FM index implementation with a suffix array sampling rate of 32).

GenMap is also suitable to compute the frequency of larger and more repetitive genomes than the human genome. We computed the (50, 2)-frequency of the barley genome (Mascher *et al.*, 2017) as it contains large amounts of repetitive DNA (Ranjekar *et al.*, 1976). Barley has 4.8 billion base pairs while the human genome has 3.2 billion base pairs. As expected, the human genome has considerably more unique regions than the barley genome. To be precise 75.4% of the 50-mers are unique in the human genome, and only 26.4% in the barley genome. There are 12.0% (54.4%), 7.6% (42.1%) and 4.8% (25.6%) 50-mers in human DNA (resp. barley DNA) with at least 10, 100 and 1000 occurrences. Computing the (50, 2)-frequency of barley on 16 threads took less than 1 h 15 m with GenMap and nearly a day with GEM using its heuristic with *t*=6 (automatically chosen by GEM).

In conclusion, GenMap is a magnitude faster than GEM in its exact mode, and still faster than GEM using its heuristic, while GenMap is always exact. Even for up to 4 errors, GenMap achieves a reasonable running time. This is due to the three techniques described in the previous section. Further improvements can be implemented which might speed up the algorithm even further, such as in-text verification (Pockrandt, 2019), i.e. locating partially searched *k*-mers and verifying whether their locations in the text match the *k*-mer with respect to the error bound. A location and verification step in the text is often several times faster than finishing an index-based approximate search.

All tests were conducted on Debian GNU/Linux 7.1 with an Intel Xeon E5-2667v2 CPU. To avoid dynamic overclocking effects in the benchmark, the CPU frequency was fixed to 3.3 GHz. The data were stored on a virtual file system in the main memory to avoid loading it from disk during the benchmark which might affect the results due to I/O operations.

We used the only available version 1.759 beta of the GEM suite that included the mappability algorithm. We did not reach the authors for other versions including their method. Other available and newer versions do not offer this feature anymore. The running times we measured for GEM heuristic differ considerably from the running times for GRCh37 published by the authors. Even when we ran it on a similar CPU with the same number of cores, we were 2–5 times slower than their published benchmarks. One reason might be that the only available version of GEM with the mappability functionality was published as a beta version; however, it was a year after their paper. Nonetheless, GenMap is still faster than the running times published by Derrien *et al.* For a fair comparison in our benchmark, we reduced the genomes to the dna4 alphabet, i.e. replaced Ns by random bases. Based on tests, we observed that GEM neither computes the mappability of *k*-mers that have unknown bases nor considers them as mismatches in its default mode even when errors are allowed.

A more recent tool to compute the mappability is Umap (Karimzadeh *et al.*, 2018). It is limited to computing the (k,0)-mappability and reporting only unique *k*-mers, i.e. regions with a mappability value of 1. It uses the read mapper Bowtie to search every single *k*-mer in the genome and filter non-unique *k*-mers afterwards. Due to these constraints, we excluded it from our benchmarks. From the authors’ benchmarks, we can conclude that GenMap still outperforms Umap as GenMap needs less than 1 h without parallelization to compute the (k,0)-mappability (see Table 1), while Umap needs about 200 h.

To verify our tool, we compared the results to an exhaustive search with Bowtie1 (Langmead *et al.*, 2009) by mapping every *k*-mer to all its possible locations. From the number of mappings of each *k*-mer, the mappability can be computed and written to a bed file. This approach yields identical results. We tested it by computing the (20, 1)-mappability on an Escherichia *coli* genome (https://github.com/cpockrandt/genmap/blob/master/tests/bowtie-test.sh) Locating all mapping positions of each *k*-mer with a read mapper would be too inefficient on eukaryotic genomes.

### 3.2 Experiments

Although the main focus of this work lies on presenting a new and fast algorithm for computing the mappability of a genome, we propose an application to identify marker sequences illustrated by a small example on *E.coli* strains.

GenMap has an option to compute the mappability on multiple genomes while at most one approximate occurrence for a *k*-mer is counted for each genome. This allows us to quickly identify *k*-mers that are unique to a genome (regardless of the overall number of approximate occurrences in this genome) or *k*-mers that occur in every genome at least once. Additionally, GenMap not only outputs the mappability or frequency but also outputs the locations where the approximate matches for each *k*-mer occur into a csv file. This helps to find marker sequences or to select candidates for probe design by identifying *k*-mers that are unique to a genome or that are present in all genomes while allowing for errors.

Marker genes or marker sequences are short subsequences of genomes whose presence or absence allows determining the organism, species or even strain when sequencing an unknown sample or helping building phylogenetic trees (Patwardhan and Ray, 2014). Depending on the marker length, it can span up to dozens of reads. Instead of assembling the strain to search for marker genes or applying experimental methods such as PCR-based amplified fragment length polymorphism (see Vos *et al.*, 1995), we propose using its mappability.

When searching for marker sequences we consider two use cases: on the one hand, we want to identify *k*-mers that match a sequence uniquely to determine the exact strain. On the other hand, we want to search for *k*-mers shared by many or all strains in the same phylogenetic group.

To test this approach, we used a dataset of *E.coli* strains. It was shown that *E.coli* can be grouped into four major phylogenetic groups (A, B1, B2 and D) (see Clermont *et al.*, 2000). The authors identified two marker genes (chuA and yjaA) and an anonymous DNA fragment (TspE4.C2) whose combination of presence or absence in the genome can determine the phylogenetic group.

**Fig. 4.**
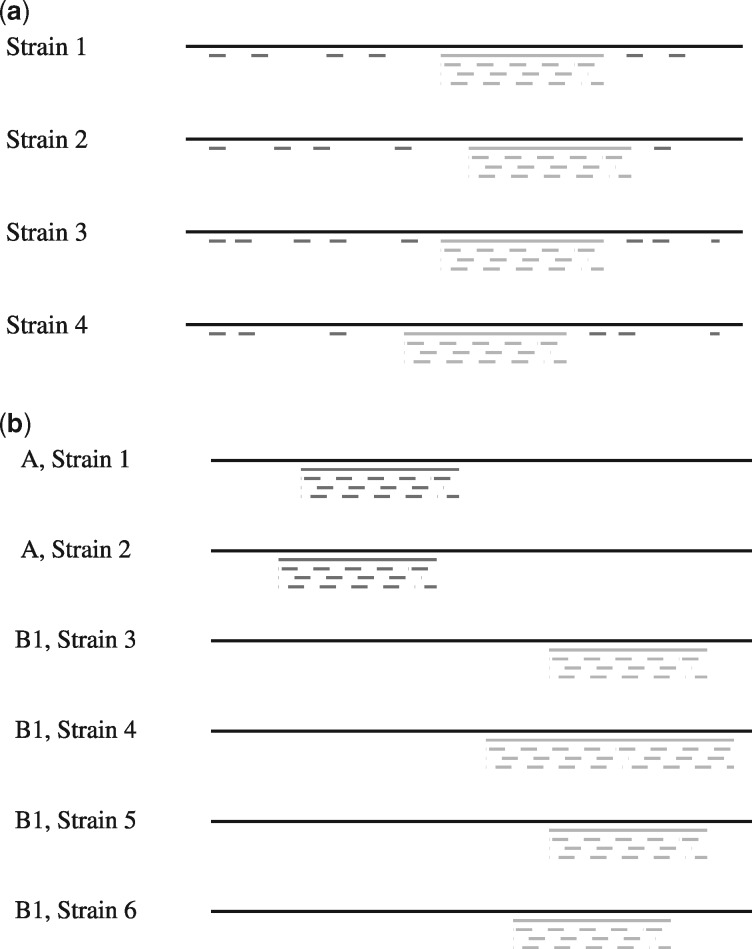
Illustration of the experiments performed on *E.coli* sequences in Tables 2 and 3. (**a**) Four strains belonging to the same phylogenetic group. The sequence in light gray is conserved within this group and a marker sequence. The light gray *k*-mers belonging to this marker sequence are also all found in the other strains. The *k*-mers in dark gray are unique among all four strains and allow distinguishing each of the strains. (**b**) Six sequences belonging to two different phylogenetic groups. Marker sequences are highlighted in light and dark gray. They only occur in one of the groups and are present in all of its strains

We computed the (30, 2)-mappability on four different strains of group B1. [Strains: IAI1 O8 (GCA_000026265.1), SE11 O152: H28 (GCA_000010385.1), 11128 O111: H- (GCA_000010765.1), 11368 O26: H11 (GCA_000091005.1).] According to the study, all strains within B1 share the anonymous DNA fragment TspE4.C2 of 152 base pairs. We used GenMap to search for both, unique *k*-mers among all strains as well as *k*-mers that occur in each strain at least once, see Figure 4a for an illustration. We observed that TspE4.C2 is an exact match in all strains and the 30-mers in this region also have a mappability value of exactly 0.25 when accounting for 2 errors. We further found numerous 30-mers with a mappability of 1, thus allowing to determine a strain among those four, while still accounting for sequencing errors and mutations. Table 2 lists the number of *k*-mers identified. We counted the number of *k*-mers matching only one strain, i.e. the strain the *k*-mer originated from. We refer to this count as *unique*. Additionally, we counted how many of these *k*-mers matched multiple times to the strain, referred to as *pseudo*. GenMap allows to exclude these pseudo marker sequences when computing the mappability on multiple sequence files, i.e. it is only counted in how many sequence files a *k*-mer is present. To avoid counting highly overlapping *k*-mers in large unique regions, we break down the numbers for non-adjacent *k*-mers as well, i.e. for a *k*-mer to be considered it must have a preceding *k*-mer with a mappability value smaller than 1.

In Table 3, we present the data of a second experiment, where we select strains from more than one group (A and B1); see Figure 4b for an illustration. Again, we computed the (30, 2)-mappability, but this time we counted *k*-mers that match all strains in one group but no strain in the other group.

**Table 3.** (30, 2)-mappability on six strains of *E.coli* of the groups A and B1

		All *k*-mers		Non-adjacent *k*-mers	
Group	Strain	Unique	∅ Dist.	Unique	∅ Dist.
A	W3110	109 375	41 ± 731	2398	1867 ± 4577
A	HS	111 179	39 ± 709	2414	1796 ± 4471
B1	IAI1	125 042	37 ± 680	3063	1485 ± 4091
B1	SE11	127 302	38 ± 690	3123	1510 ± 4148
B1	11128	121 325	42 ± 766	3275	1548 ± 4408
B1	11368	131 121	41 ± 814	3473	1537 ± 4763

*Note*: Only *k*-mers were counted that perfectly separated the strains in A from B1, i.e. if and only if the *k*-mer matched all strains of A and no strain of B1 and vice versa.

This example shows that mappability on multiple species or strains can be used to identify possible marker sequences. Short *k*-mers could be used to search a dataset of reads instead of searching for marker genes that span multiple reads. Since computing the (30, 2)-mappability on a few *E.coli* strains even takes less than a minute on a consumer laptop, this method is suitable to be run on large sets of similar *E.coli* strains to identify new marker sequences, even with errors accounting for uncertainty arising from sequencing and mutations such as SNPs.

## 4 Discussion

We have presented GenMap, a fast and exact algorithm to compute the mappability of genomes up to *e* errors, which is based on the C++ sequence analysis library SeqAn (Reinert *et al.*, 2017). It is significantly faster, often by a magnitude than the algorithm from the widely used GEM suite while refraining from heuristics.

Mappability has already been used for various purposes (Derrien *et al.*, 2012). In this paper, we proposed a new application, the computation of mappability on a set of genomes to identify marker sequences for grouping and distinguishing genomes by short *k*-mers and illustrated it with a small example on closely related *E.coli* strains. It is also suitable for large scale data as demonstrated by the benchmarks on human and barley genomes in Section 3.1.

The ability to compute the mappability efficiently opens up new applications such as incorporating the mappability information into the read mapping process itself instead of the post-processing phase. During the index-based search of a read, the possible locations of the eventually completely mapped read can be examined beforehand to filter repetitive regions without repeat masking. This allows for new mapping strategies to improve the running time of state-of-the-art read mappers and reduce post-processing overhead (Pockrandt, 2019).
